# Giant asymptomatic left renal oncocytoma in a 40-year-old man: a case report

**DOI:** 10.11604/pamj.2022.42.177.35965

**Published:** 2022-07-05

**Authors:** Sultan Qaid, Radman Ghaleb, Faisal Ahmed, Ebrahim Al-shami, Qasem Alyhari, Saleh Al-wageeh, Mohammad Reza Askarpour

**Affiliations:** 1 Urology Research Center, Al-Thora General Hospital, Department of Urology, School of Medicine, Ibb University of Medical Science, Ibb, Yemen,; 2 Department of Urology, Alhamd Hospital, Ibb, Yemen,; 3 Department of General Surgery, School of Medicine, Ibb University of Medical Science, Ibb, Yemen,; 4 Department of Urology, School of Medicine, Shiraz University of Medical Sciences, Shiraz, Iran

**Keywords:** Giant, renal oncocytoma, central stellate scar, case report

## Abstract

Renal oncocytoma is a benign tumor that arises from epithelial cells of the distal renal tubules. It is naturally presented with a small-sized mass, and giant oncocytoma is uncommon. Renal oncocytoma is frequently asymptomatic and challenging to distinguish preoperatively from renal cell carcinoma (RCC). We present a 40-year-old man who presented with intermittent abdominal pain in the last two years. Abdominal computed tomography (CT) scan showed a large, heterogenous left renal mass measured 15 x 16 x 19.5 cm and associated with central calcifications suspected of RCC. The patient underwent a left radical nephrectomy without complication. The histopathological study revealed typical oncocytoma features. There was no detected recurrence or distant metastasis on six months follow-up. In conclusion, it is challenging to distinguish renal oncocytoma from RCC via preoperative radiology images, especially when a giant mass is present. The only histopathology examination of the removed specimen can provide a definitive diagnosis.

## Introduction

Renal oncocytoma is responsible for 3-7% of all kidney cancers. The average age of onset is in the sixth-seventh decades with male predominance. Renal oncocytoma is primarily single and unilateral, but in 4-5% of cases, it is bilateral, and in 13%, it might be multifocal [1]. Renal oncocytoma is typically hypervascular and homogeneous and presents a characteristic central scar on computed tomography (CT) scan. However, these radiological findings had a poor predictive value and no definite radiologic features to differentiate it from renal cell carcinoma (RCC) [2]. Oncocytoma is naturally presented with a small-sized mass, and giant oncocytoma is uncommon. Nevertheless, the prognosis of enormous oncocytoma is similar to other smaller lesions [3,4]. Few patients reported incidentally detected giant renal oncocytomas in the literature [2,3]. Therefore, we reported a case of huge left renal oncocytomas in a 40-year-old man.

## Patient and observation

**Patient information:** a 40-year-old male presented with generalized abdominal pain for two years. The pain was mild and radiated to the left flank area. No history of urinary tract symptoms such as hematuria or dysuria. No record of fever, weight loss, or other constitutional symptoms. The patient was a nonsmoker without a family history of cancer.

**Clinical findings:** the patient´s vital sign was stable, and a palpable abdominal mass in the left upper quadrant was detected on physical examination. The mass was not mobile or tender.

**Figure 1:**
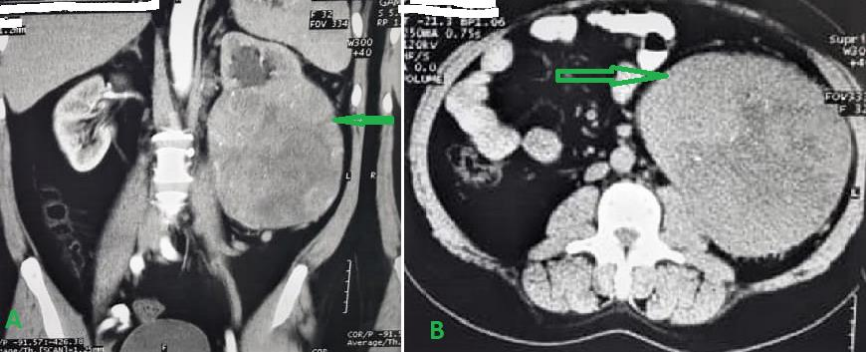
abdominal computed tomography scan showing the left renal mass with solid and cystic composition in axial (A) and coronal (B) views (arrows)

**Diagnostic assessment:** liver and renal function tests, and haematological tests, are normal (Routines Lab tests). The ultrasonography (US) of the abdomen showed a large, well-defined, varied echogenicity and central necrosis left renal mass measured about 15 x 16 cm. A computed tomography (CT) scan of the abdomen with intravenous contrast administration showed a large, heterogenous left renal mass measured about 15 x 16 x 19.5 cm. The mass was well-defined, lobulated, and associated with central calcifications. Radiological characteristics were insufficient to distinguish this lesion from RCC (Figure 1).

**Figure 2:**
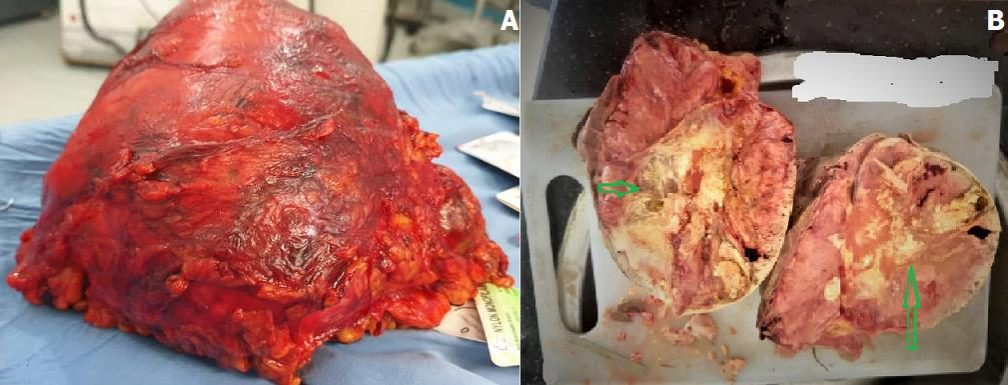
intraoperative photo of mass showing a giant resected mass (A) and central area of scarring (arrows) (B)

**Therapeutic interventions:** after general anesthesia and with a left subcostal incision, the retroperitoneal space was opened. A large renal mass was identified. After mobilization and release of the left kidney, radical nephrectomy was performed. The specimen weighed 3500 g and measured 15 x 16 x 195 cm (Figure 2).

**Figure 3:**
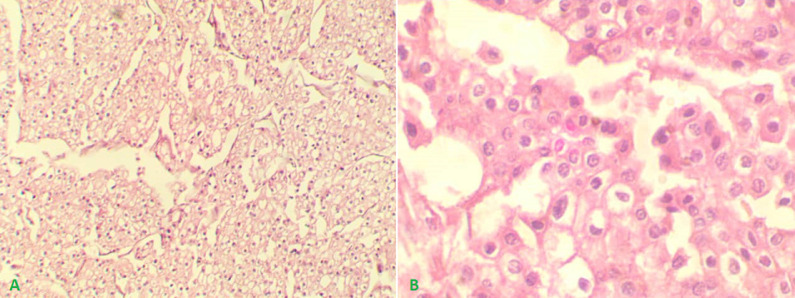
histopathologic examination showing the classic architecture of an oncocytoma; large eosinophilic cells arranged in distinct nests (A x100, B x200)

**Follow-up and outcome:** the patient´s postoperative recovery was unremarkable, and he was discharged without complications on the third postoperative day. The histopathology showed tumour cells formed of sheets and alveoli of mosaic oncocytic cells with small rounded nuclei, sometimes binucleation with perinuclear halo and low nuclear/cytoplasmic ratio (features between oncocytoma and chromophobe renal cell carcinoma). No capsular invasion or renal sinus invasion. The final diagnosis was oncocytic renal neoplasm of low malignant potential, stage II (pT2bNxMx) (Figure 3).

**Patient perspective:** during treatment, the patient was satisfied with the level of care provided to him.

**Informed consent:** written informed consent was obtained from the patient for participation in our study.

## Discussion

Oncocytomas are the most common benign solid renal tumours, usually misdiagnosed preoperatively with RCC, and definite diagnosis usually obtained via histopathologic evolutions [5]. The current case describes a rare asymptomatic giant renal oncocytoma that was difficult to differentiate from RCC preoperatively depending on the radiologic features of the CT scan. Zippel *et al*. firstly described this tumor as a distinct pathologic entity in 1942 [3]. As in our case, these lesions are usually asymptomatic and discovered incidentally during a workup for another reason. However, hematuria, palpable mass, and flank pain are the most frequent complaint in symptomatic patients [2].

The US, CT scan, and magnetic resonance imaging are helpful radiological imaging techniques for diagnosis. Oncocytomas appear on CT scans as a solid mass with homogeneous and varying attenuations, similar to RCC. The central stellate pattern scar on the CT scan could indicate the diagnosis of oncocytoma. However, CT scans still had inadequate predictive value and could not accurately distinguish oncocytomas from RCC [4]. In our case, the central scar was seen on the CT scan; however, the CT scan was still suspicious for RCC. Fine-needle aspiration (FNA) may provide a preliminary diagnosis. However, it is not reliable for accurate diagnosis. In FNA biopsy, no sufficient tumour specimen is obtained, and the risk of bleeding from a hypervascular tumour may occur. Additionally, RCC and oncocytoma may present in the same lesion or different areas of the same kidney [6]. Due to the preoperative suspicion of RCC and the unreliability of frozen section diagnosis, radical nephrectomy is the least risky treatment unless other factors preclude it, such as solitary kidney, bilateral tumours, or poor renal function [6].

Oncocytomas are commonly associated with three distinct genetic abnormalities; nevertheless, no chromosomal abnormalities have been found in several cases. The genetic associations are chromosome Y loss or monosomy, translocations in the 11q13 region, and congenital abnormalities such as trisomy, monosomy, or heterozygosity loss [7]. These genetic changes are unique to oncocytoma and do not occur in RCC [7]. There were no congenital abnormalities in our patient. Radical nephrectomy is still the gold standard treatment for giant renal oncocytomas [3]. However, surgical treatment of small masses is still unclear. Organ-sparing surgery such as partial nephrectomy should be kept for bilateral tumours, tumours less than 4 cm in upper or lower poles, or solitary kidney patients [5]. Oncocytomas and different histological subtypes of RCC can usually be distinguished based on gross and microscopic histopathologic examination of removed spacemen. However, sometimes distinguishing it from the eosinophilic variant of chromophobe RCC (ChRCC) and the granular variant of conventional RCC is difficult and Immunohistochemistry (IHC) makes an accurate final diagnosis [8]. In our case, gross and microscopic histopathologic examinations of removed spacemen were enough to make the definitive diagnosis.

The most useful IHC markers are Vimentin, CK7, DOG1, Cyclin D1, and CD10. In Vimentin which is (+) in conventional RCC, (-) ChRCC, and (-) in oncocytoma. CK7 which (+) in ChRCC, (-) in oncocytoma, and (-) in conventional RCC. CD10 which (+) in conventional RCC, (-) in ChRCC, and (-) in oncocytoma. DOG1 was positive in ChRCC and renal oncocytoma and negative in RCC. Cyclin D1 was positive in renal oncocytomas but negative in the ChRCC and RCC. Hale´s colloidal iron staining with diffuse reticulitis which (+) in ChRCC but (-) in oncocytoma and (-) in conventional RCC [8,9]. Renal oncocytoma is an almost benign tumour, with no reports of metastasis or recurrence, even giants in size. In 10% to 32% of patients, the tumor could coexist with RCC or have rapid growth and even destroy adjacent parenchyma, demanding close monitoring of the tumor for possible mandatory intervention [3,4]. In our case, there was no recurrence or metastasis in the first six months after surgery. Few cases of giant oncocytomas have been reported, such as Demos *et al*., who reported oncocytoma measured 27 × 20 × 15 cm and weighed 4652 g [10]. Sundararajan *et al*. reported renal oncocytoma weighted 3353 g and sized 20 cm [11]. Banks *et al*. reported renal oncocytoma weighted 3090 g and sized 21 x 18 x 15 cm [12]. Akbulut *et al*. reported renal oncocytoma weighed 3380 g and measured 25 x 15 x 12 cm [2]. Similar to the previous reports, our case was a giant renal oncocytoma that weighed 3500 g and measured 15 ×16 ×19.5 cm.

## Conclusion

Renal oncocytoma disregarding the size has a good prognosis and a benign clinical course. Unfortunately, clinical or radiographic criteria cannot differentiate most renal oncocytomas from malignant RCC. In larger renal masses, such as our case, radical nephrectomy will remain the preferred management strategy.
